# Chromatin’s Influence on Pre-Replication Complex Assembly and Function

**DOI:** 10.3390/biology13030152

**Published:** 2024-02-27

**Authors:** Hina Ahmad, Neha Chetlangia, Supriya G. Prasanth

**Affiliations:** 1 Department of Cell and Developmental Biology, University of Illinois at Urbana-Champaign, 601S Goodwin Avenue, Urbana, IL 61801, USA; hinaa2@illinois.edu (H.A.); nehac4@illinois.edu (N.C.); 2 Cancer Center at Illinois, University of Illinois Urbana-Champaign, Urbana, IL 61801, USA

**Keywords:** DNA replication, chromatin, ORC, pre-replication complex (pre-RC), epigenetics, polycomb (Pc)

## Abstract

**Simple Summary:**

Genome duplication and the faithful segregation of chromosomes to daughter cells are crucial for the maintenance of genome stability. Replication initiators play a crucial role in ensuring that DNA replication occurs only once per cell division cycle. This review summarizes when, where, and how replication initiates in human cells.

**Abstract:**

In all eukaryotes, the initiation of DNA replication requires a stepwise assembly of factors onto the origins of DNA replication. This is pioneered by the Origin Recognition Complex, which recruits Cdc6. Together, they bring Cdt1, which shepherds MCM2-7 to form the OCCM complex. Sequentially, a second Cdt1-bound hexamer of MCM2-7 is recruited by ORC-Cdc6 to form an MCM double hexamer, which forms a part of the pre-RC. Although the mechanism of ORC binding to DNA varies across eukaryotes, how ORC is recruited to replication origins in human cells remains an area of intense investigation. This review discusses how the chromatin environment influences pre-RC assembly, function, and, eventually, origin activity.

## 1. Introduction

Eukaryotic cells need to replicate their genome accurately, which requires carefully orchestrated molecular players acting at the right time and place. Replication starts at replication origins, where a sequence of binding events occurs [1]. At the end of mitosis/early G1, the Origin Recognition Complex (ORC) assembles at these replication origins. ORC recruits Cdc6 (cell division cycle 6) and Cdt1 (chromatin licensing and DNA replication factor 1), which helps to load the minichromosome maintenance protein complex (MCM complex consisting of MCM2-7) to form the OCCM complex. The recruitment of a second MCM2-7 hexamer to form the head-to-head hexamers completes the pre-RC assembly (reviewed in [2,3]). Origin licensing, which involves the loading of double hexameric MCM complexes at replication origins, is a highly conserved process. This process ensures that DNA replication occurs only once per cell division cycle.

Our understanding of the intricate steps of replication initiation comes from elaborate work in budding yeast, though many of the principles are also conserved in higher eukaryotes. The initiation of replication is further controlled by Dbf4-dependent kinase (DDK) and cyclin-dependent kinase (CDK), which are sequentially activated during the transition from G1 to the S phase [4,5,6,7,8]. DDK phosphorylates MCM2-7 subunits, which allows the loading of Cdc45 and Sld3, followed by the phosphorylation of Sld2 and Sld3 by CDK [9,10,11,12,13,14]. A series of events ensures that the CMG complex (Cdc45, MCM2-7, and GINS) is activated as the helicase for replication to proceed [15].

The origin recognition complex (ORC) was identified from cell extracts of Saccharomyces cerevisiae as a heterohexameric complex bound to the autonomously replicating sequence (ARS, origins of replication in Saccharomyces cerevisiae), comprising Orc1 through to Orc6 in descending order of their molecular mass [16]. Orthologs of each ORC subunit have been identified in diverse organisms, including humans, frogs, flies, nematodes, and plants [17]. Although the replication origins vary from one species to another, ORC binds to replication origins in diverse organisms. What defines an origin and how ORC recognizes these origins differs significantly amongst different species.

**Figure 1:**
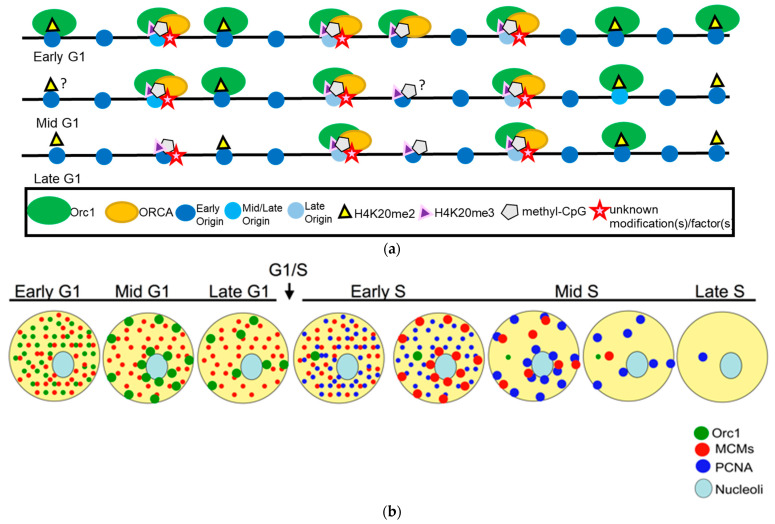
(**a**) The temporal association of Orc1 to H4K20me2 and ORCA to H4K20me3 during G1 dictates early and late origins in humans. Live cell imaging, immunofluorescence, and genome-wide ChIP studies have demonstrated the spatiotemporal localization of Orc1 and ORCA during the cell cycle. Time-lapse imaging studies predict that human Orc1 associates with all origins in early G1 and then is sequentially released from most sites that represent heterochromatic sites, except at late origins. Orc1 associates with H4K20me2/3 marks. ORCA is associated with repressive histone marks, primarily H3K9me3 and H4K20me3, and is likely enriched only at late-firing origins in G1. (**b**) Model depicting organized patterns of Orc1 during the G1 phase and how it anticipates the spatiotemporal dynamics of MCMs and PCNA in sequential order. During early G1, Orc1 is present as punctate foci throughout the nucleus, and as cells progress through G1, it is enriched only at heterochromatic sites. The MCM complex shows similar patterns to Orc1, but it is homogenously distributed in G1, shows temporal patterning during the S phase, and is lost from most sites by the end of the S phase. Finally, PCNA shows punctate foci in the early S phase and is redistributed to heterochromatic sites during the mid and late S phase.

In human cells, Orc2-5 forms the core of the ORC and remains associated as a complex throughout the cell cycle [18,19,20,21]. Several years ago, our laboratory identified ORC-Associated/Leucine-rich and WD-repeat-containing (ORCA/LRWD1) as an ORC-associated factor that stabilized ORC onto chromatin [22,23,24]. The core-ORC and ORCA are stably associated with one another throughout the cell cycle. Human ORC subunits are highly dynamic in their association with chromatin across the cell cycle. Orc2 and ORCA are released from most chromatin sites at the end of G1, but remain associated with heterochromatin sites, including telomeres and centromeres in human cells [25,26,27]. The largest subunit, human Orc1, is spatiotemporally regulated (Figure 1) and is dynamically associated with the ORC on the chromatin and is degraded at the end of G1 following SCF/Skp2-mediated ubiquitination [28]. It is reloaded onto the chromatin during the establishment of the next pre-RC in the late M to early G1 phase [29]. Multiple ORC subunits belong to the AAA+ family of proteins. ORC is an ATPase, and its binding to the origin DNA is ATP-dependent [30].

## 2. Chromatin Signature at the Origins

In metazoans, replication origins are highly heterogeneous, and their regulation is complex [31]. Replication in metazoans initiates at sites that do not show any clear sequence specificity, unlike in budding yeast, where the initiation sites are, to a large extent, clearly defined [32]. Recent analyses have suggested that GC-rich sequences, which are origin G-rich repeated elements (OGREs) that can form G4 quadraplexes, transcription start sites, and regions of DNAse hypersensitivity, are often found at metazoan origins [33,34,35,36,37,38,39,40,41,42]. Replication initiates in moderately transcribed regions and is associated with DNase hypersensitive sites. Moreover, genome-wide experiments have bolstered the co-regulation model of replication initiation and transcription [43]. These are active areas of study, and we hope to learn the mechanistic details in the coming years.

In higher eukaryotes, the DNA sequence is not the sole determinant of replication initiation events, and origin specification may depend on epigenetic features. The firing of origins is spatially and temporally controlled in a cell-, tissue-, and development-specific manner [37,44,45,46,47,48]. The stochastic model of replication origin firing posits that the firing time of an individual origin in a population of cells is heterogeneous, firing early in some and late in others. The heterogeneity of the stochastic model makes identifying origins challenging. The Dutta and Zang labs have recently suggested that the origins are specified by diverse stochastic events that are dependent on the epigenetic accessibility around promoters. However, intriguingly, the ORC-binding sites or the MCM complex did not overlap with the origins [49]. A series of genome-wide studies with various ORC antibodies, specifically at distinct time points within G1, would be important to pinpoint ORC dynamics in human cells.

It has been suggested that the chromatin environment plays a crucial role in dictating the activation of replication origins [37]. A common feature of all yeast origins seems to be a nucleosome-free and G-rich region that is present upstream of a nucleosome positioned at the initiation sites. These results support the concept of chromatin-dependent origins in budding yeast [50]. The polycomb group (PcG) of proteins comprises two multi-subunit complexes, PRC1 and PRC2, catalyzing H2AK119ub and H3K27me3, respectively. PcG proteins are involved in transcriptional silencing. For example, the association of H3K4me1 with H3K27methylation, catalyzed by the polycomb group protein EZH2, is suggested to provide an environment conducive for DNA replication initiation [51]. Supporting work has shown that PcG proteins are required for origin activation [52,53] and that these proteins structurally restrict origin activation within the polycomb domains. The chromatin mark H3K4me3, which is associated with active transcription and open chromatin, is often found at the origins of mouse stem cells. These origins also have H3K9ac, facilitating accessibility [54]. It is generally accepted that chromatin-modifying factors can create a chromatin environment that enables transcription and replication [55,56,57].

Comprehensive work from the Aladjem lab has demonstrated that common origins are associated most strongly with unmethylated CpG islands, H3K4me3, and H3K9Ac. In contrast, cell-type-specific origins are primarily associated with methylated CpG islands and H3K9me3 and replicate late [42]. They further demonstrated that the early replicating regions often correlate with histone marks associated with transcriptionally active domains, such as H3K4me1/2/3, H3K9ac, H3K18ac, H3K36me3, and H2K27ac. In contrast, late replicating origins are associated with heterochromatin, are often associated with H3 and H4 hypoacetylation, and are enriched with H3K9 and H3K27 methylation [36,37,42]. A list of the various chromatin marks associated with various aspects of replication initiation are listed in Table 1.

## 3. Chromatin Governs the Spatiotemporal Dynamics of Pre-RC Factors

Accumulating evidence points towards the fact that global chromatin structures dictate the replication program, starting from the establishment of pre-RC to the initiation of replication [58]. We have demonstrated that human Orc5 associates with the histone acetyltransferase and GCN5. Tethering Orc5 to a chromatin locus increases acetylation-mediated large-scale decondensation [59]. The ability of Orc5 to induce chromatin unfolding during G1 likely enables the establishment of pre-RC at the origins. Similarly, H4 acetylation at the origins by the ORC-interacting protein HBO1 (human acetylase binding to ORC1) is critical for replication licensing by Cdt1 [60,61]. ORC-mediated chromatin acetylation has been suggested to control DNA replication through pre-RC formation [62].

**Figure 2:**
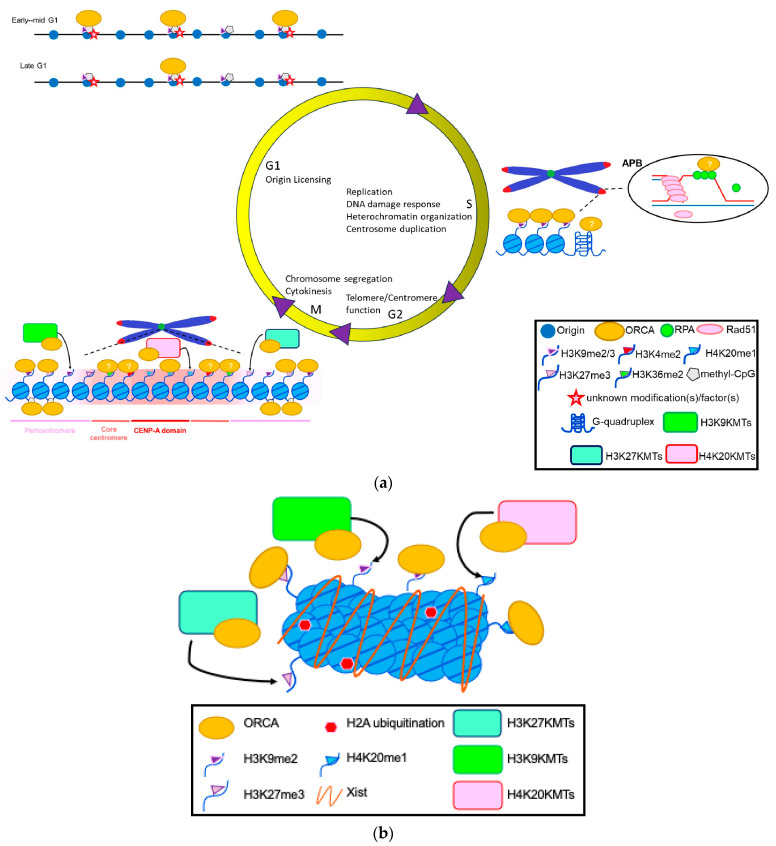
(**a**) Replication initiators controlling diverse cellular functions, coordinating the completion of duplication with chromosome segregation and cell division. At origins: a model depicting the binding of ORCA to specific replication origins enriched for H3K9me3. At telomeres: the binding of ORCA to ALT-telomeres could be through binding to H3K9me3 and G-quadruplex DNA through associations with RPA. APB: ALT-associated PML body. At centromeres: the binding of ORCA to various chromatin marks that represent origins or for mitotic regulation. (**b**) At facultative heterochromatin: the binding of ORCA to an inactive X-chromosome. The figure models have been adapted and modified from Wang et al., 2017 and Giri et al., 2015 [64,67].

ORC and ORCA/LRWD1 associate with repressive histone marks, namely H3K9me3, H4K20me3, and H3K27me3 (Figure 2) [63,64,65,66,67]. Recent cryoEM work from the Bleichert lab has suggested that ORCA establishes a ternary complex by simultaneously recognizing Orc2, nucleosomal DNA, and repressive histone trimethylation through an aromatic cage [68]. The authors suggest that this enables reorganization of the local chromatin architecture by diminishing nucleosome self-association. Interestingly, ORC and ORCA also bind to methylated DNA and the methyltransferases that catalyze the above histone modifications and DNA methylation. Our results have previously demonstrated that ORCA promotes late replication, similar to the ORC-mediated stabilization of heterochromatin protein 1 (HP1 α and β in human cells) for establishing late replicating domains [69,70,71,72]. This is supported by new evidence that ORCA is necessary and sufficient to recruit ORC into chromatin condensates marked by H4K20 trimethylation [68]. Although increasing evidence suggests that ORCA plays a key role in recruiting ORC to specific chromatin sites, it is equally intriguing that Orc1 binds to H4K20me2 at the origins via its bromo-adjacent homology (BAH) domain [73]. Further, the loss of this association results in failure of Orc1 to localize to the origins [73,74]. These results support that ORC/ORCA binding to specific chromatin marks is important for the spatiotemporal regulation of DNA replication (Figure 1 and Figure 2).

The histone mark H4K20me1 has also been shown to be present at some human DNA replication origins [75,76,77,78]. Specifically, tethering PR-Set7, the methylase responsible for H4K20 methylation, to a specific genomic locus promotes pre-RC assembly. PR-Set7 is degraded by the CRL4-Cdt2 ubiquitin ligase complex during the S phase of the cell cycle and, accordingly, the stabilization of PR-Set7 or depletion of Cdt2 promotes re-replication. This re-replication phenotype depends on the subsequent trimethylation of H4K20 by Suv4-20h1/h2. Other methylation marks, such as H3K4me2 and me3, are also found at replication origins, but their function still needs to be determined.

Nucleosomes containing the H2A.Z variant can recruit Suv420H1, which is a methyl transferase, that, in turn, facilitates the deposition of H4K20me2, which is eventually recognized by the BAH domain of Orc1 [79]. The genome-wide depletion of H2A.Z leads to decreased H4K20me2 and Orc1 and nascent strand signals throughout the genome. H2A.Z-regulated replication origins fire early and have higher firing efficiency. Nucleosome-free regions next to the location of H2A.Z facilitate pre-RC loading. Although H2A.Z at the promoters seems to be required for epigenetically regulating licensing, the exact mechanism remains to be deciphered. Recently, a new functional genetic element, pG4s, associated with specific nucleosome-free regions (NFRs), was found to be sufficient for replication initiation. The authors suggested that this might be a strategy for the ORC recognition of origins in eukaryotes [80].

Work from the Liu lab has shown that yeast ORC has intrinsic nucleosome remodeling activity, which can evict H2A-H2B dimers, leaving the 2(H3-H4) tetramer on DNA, and this requires the Orc1-BAH domain [81,82]. Exciting work from the Kurat lab has recently established that in yeast, Orc1 collaborates with chromatin remodeling complexes, including INO80, ISW1a, ISW2, and other chromatin modifiers, to establish a Nucleosome Free Region (NFR) and a flanking nucleosomal array at yeast origins [83]. The theme that ORC binding sites are found at NFRs upstream of the transcription start site (TSS) seems to be conserved amongst eukaryotes [60,84,85,86,87,88]. This has provided novel insights into the critical role of ORC in nucleosome organization at the replication origins.

Human Orc1 is intriguing because it is one of the only known proteins to show dynamic spatiotemporal patterning during the G1 phase of the cell cycle (Figure 1b) [29]. Whether Orc1 binds to specific chromatin signatures during different stages within G1 to dictate MCM complex loading or sets the marks where MCM2-7 and finally PCNA would load remains an open question (Figure 1b). In budding yeast and metazoans, Orc1 has an intrinsically disordered region (IDR) that facilitates binding to the minor groove of origin DNA. A domain within the human Orc1 IDR is required for interaction between the Orc1 and CDC6 AAA+ domains in G1 [89]. The IDRs of DNA replication initiators have been shown to drive DNA-dependent phase separation in vitro and chromosome binding in vivo, and these initiator condensates selectively recruit replication-specific partner proteins for the initiation of DNA replication [90].

## 4. Chromatin Dictates Origin Activation

The acetylation of histones has been linked to open chromatin, and dynamic changes of this modification are known to regulate replication origin activation in yeast [91]. Also, H4K16 deacetylation decreases MCM complex loading onto early origins, dramatically changing the licensing landscape [92,93]. Similarly, at the rDNA locus, the histone deacetylase (SIR proteins) represses a large majority of the replication origins. This epigenetic silencing of origin firing promotes genome stability [94]. SIR proteins can act at the rDNA locus, euchromatic origins, and telomeres [95,96,97]. An increase in the local concentration of histone acetylation has been shown to improve Cdc45 recruitment [98]. Constitutive H3 K56ac sensitizes cells to replicative stress, in part, by negatively influencing the activation of the origins of DNA replication [99].

The methylation of histones has a varied impact on replication origin function. The lack of the ubiquitous mark H3K37me1 from origins facilitates MCM2-7 binding to origins because the presence of this mark impedes MCM2-7 association with chromatin; however, the mechanism is not known [100]. H3K4 di-methylation was found to be important for origin function [101]. Further, H3K36 methylation has been found to regulate the timing of Cdc45 binding to origins, whereas mono-methylation is associated with early replication origins and trimethylation is present at late origins [102].

The chromatin architecture, including the nucleosome positioning and occupancy at origins, impacts origin specification and activation. How this affects ORC and MCM complex loading and what kind of histone marks dictate whether an origin is permissive or restrictive to origin activation remains to be elucidated [103]. This would require a high genome-wide distribution of histone marks during temporally defined windows in G1. An AI-based tool could pinpoint whether a specific signature dictates specific origins and their efficiency, including what has been done to identify the splicing-associated chromatin signatures [104].

## 5. Euchromatin vs. Heterochromatin: The Replication Timing Issue

Are specific chromatin marks in the human genome dictating replication origin specification and activity? This is a conundrum that has been an active area of investigation. The consensus is that the presence of active marks associated with open chromatin and transcriptional activity, H3K4 methylation, and histone acetylation (H3K9ac, H3K18ac, H3K36ac, and H3K27ac) is correlated with early origin specification. Acetylation at H4K5, H4K8, and H4K12 by HBO1 and ORC-interacting factors is needed for replication licensing and facilitates MCM complex loading [60]. In contrast, the repressive histone marks H3K9 methylation and H3K27 methylation, accompanied by hypoacetylation, are at late-firing origins. These conclusions are based on correlative data, and it is likely a combination of chromatin marks, genomic context, cell-type-, tissue-type-, and developmental-context-dependent states that determines origin specification, ORC–MCM binding, and Cdc45 recruitment. For example, although H3K4me3 may be present at promoters and is likely associated with early origins, data has also suggested this mark represses origin firing. Further, the demethylase of H3K4me3, KDM5C, is needed for fine-tuning the methylation status at origins, and highly elevated levels of this mark result in replication defects due to a Cdc45 loading defects at origins [105].

Heterochromatin marks, including H3K9me3, are usually associated with late origins, and the demethylase KDM4D facilitates the formation of a pre-initiation complex and regulates DNA replication [106]. H3K27me3 is another repressive mark, and the methyltransferase EZH2 is strongly associated with a subset of origins and participates in the activity of these origins [107]. Although H4K20 trimethylation is often linked to heterochromatin sites and is bound by ORCA/LRWD1, H4K20 di-methylation is enriched at origins and is bound by ORC. Finally, the nucleosome containing the histone variant H2A.Z is enriched together with H4K20me2 and Orc1. H3K79 di-methylation is another modification enriched at origins, and the methyl transferase Dot1 is important for preventing genome re-replication [108].

**Table 1:** Histone modifications with their associated factors and sites.

Histone Modification/Marks	Downstream Effect on Transcription	Associated Factors	Sites/Functions	References
H3K37me1	Repression	ARS/Set1p/Set2p	Replication origin licensing	Santos-Rosa et al., 2021 [100]
H3K4me2	Activation	Orc1/HAT/SAGA complex	Likely at origins	Rizzardi et al., 2012, Rondinelli et al., 2015 [101,105]
H3K79me2	Activation	CAF-1, 53BP1	Replication initiation events/G1-S phase	Fu et al., 2013 [108]
H3K4me3	Activation	Data	Early replication origin/pomoter activation	Smith et al., 2016, Cayrou et al., 2015 [37,42]
H3K36me3	Activation	Set2/Cdc45 recruitment	Origin firing during S Phase/Transcription associated mark	Giri and Prasanth, 2015, Unnikrishnan et al., 2010, Smith et al., 2016 [42,65,91]
H3K9ac	Activation		Early replication origin/pomoter activation	Smith et al., 2016, Cayrou et al., 2015 [37,42]
H3K27ac	Activation	RNA pol II/Gcn5/KAT2	Enriched at transcriptionally active regions	Cayrou et al., 2015, Unnikrishnan et al., 2010 [37,91]
H3k18ac	Activation		Early replication origins	Mechali et al., 2013 [36]
H3K56ac	Activation	Rtt109	Enriched in S phase	Tremblay et al., 2023 [99]
H4K20me1	Repression		PR-Set7 to enhance pre-RC formation via H4K20me/high levels in G2/M phase	Abbas et al., 2010, Mechali et al., 2013 [36,75]
H4K20me2	Repression	ORC1/H2A.Z	Enriched at origins	Long et al., 2020, Kuo et al., 2012, Mechali et al., 2013 [36,73,79]
H4ac	Activation	Cdt1	Condensed chromosomes	Miotto and Struhl, 2010; 2011 [60,61]
H4K5, H4K8, H4K12 ac	Activation	HBO1	MCM loading	Miotto and Struhl, 2010 [60]
H4K16ac	Activation		MCM loading/Sas2	Hoggard et al., 2020, Unnikrishnan et al., 2010 [91,92]
H3K4me1 + H3K27me3	Activation + Repression		Early origins/replication initiation sites	Cayrou et al., 2015 [37]
H3K9me3, H3K27me3, H4K20me3	Repression	ORC/ORCA/HP1/PC1	Early origins/origin licensing	Pasini et al., 2004, Piunti et al., 2014, Kuzmichev et al., 2002, Gorisch et al., 2005, Bartke et al., 2010, Giri et al., 2015, Giri and Prasanth, 2015, Vermeulen et al., 2010, Wang et al., 2017 [51,52,53,54,63,64,65,66,67]

It is increasingly becoming evident that rather than individual chromatin marks, a combination of marks might dictate origin specification, usage, and function. For example, H3K4me3, H3K9me3, and H3K36me3 are likely associated with late-replicating origins. However, a recent study demonstrated that the presence of all these combinatorically resulted in an early replication phenotype. Furthermore, the addition of H3K56ac and the above three resulted in a higher likelihood of early replication [109,110].

## 6. Replication-Independent Role of ORC/ORCA in Heterochromatin Organization

ORC is implicated in transcriptional silencing in diverse eukaryotes, including yeast, drosophila, and humans [111]. In humans, the core ORC, Orc2-5, and ORCA associate with heterochromatin sites outside of G1 and bind to heterochromatin proteins HP1α and β. Furthermore, these factors play a role in heterochromatin organization. The loss of ORC and ORCA results in redistribution of the bonafide heterochromatin protein HP1α, with a concomitant alteration in the genome-wide frequency of the H3K9me3 repressive mark [64,72]. This was consistent with chromatin decondensation at satellite repeats. Using a degron-based method, we previously demonstrated that the role of human ORCA in heterochromatin organization is independent of its role in replication licensing, providing important insights into the multiple roles that these replication initiators play during the cell cycle [64]. Corroborating our data, the loss of mouse ORCA resulted in the activation of major satellite repeats, supporting the view that ORC-ORCA may play a role in silencing repeat elements [112].

The post-translation modification of ORC2 by SUMOylation was found to be required for recruiting the demethylase KDM5A. The conversion of H3K4me3 to H3K4me2 increased α-satellite transcription at the centromeres. The aberrant expression of SUMO-less Orc2 caused a reduction in α-satellite transcription and defective pericentric heterochromatin silencing, ultimately causing the re-replication of heterochromatin DNA [113]. These suggest that ORC is required to maintain genomic stability by maintaining accurate centromeric histone methylation, including H3K4, H3K9, and H4K20 trimethylation. Further experimentation is needed to establish whether the function of ORC-ORCA at the centromeric heterochromatin is related to its role in chromatin organization or the establishment of origins during mitosis (Figure 2).

All human ORC proteins are localized to constitutive heterochromatic sites, including centromeres. As suggested in a recent review, the centromeric regions and origins may have been linked in ancestral eukaryotes [1]. Centromeres may represent the origins at which ORC binds during mitosis to establish pre-RC. To support this model, it was reported that ORC is required to maintain alpha-satellite sequences at the centromere [72]. It is also important to note that repressive histone marks are enriched at centromeres, and it remains to be determined whether ORC is enriched at these sites via its interaction with H3K9me3 or H4K20me3, particularly at the pericentromeric regions (Figure 2). DNA combing experiments have suggested that the origin density is higher within the pericentromeric satellite repeats [114]. Also, the telomere repeat factor binding protein TRF2 interacts with Orc2 in pericentromeric DNA, which is required for origin activity [114]. Another possibility is the presence of H3K4 methylation, as is reported at the origins and centromeres of nematodes. With the completion of the T2T assembly of the human genome [115], we will now be able to delve deep into chromatin organization at the centromere.

ORC and ORCA also localize to centromeres and telomeres and associate with the TRF2 protein [25,116,117,118]. TRF2 binds to the origin recognition complex (ORC) and has previously been implicated in loading ORC and MCM complexes onto DNA at telomeres. Work from the Fujita lab has shown that TRF2-mediated ORC recruitment contributes to the suppression of telomere instability [118]. Its interaction with SUMOylated shelterin components facilitates ORCA’s localization to the ALT-telomeres. The loss of ORCA causes global chromatin decondensation, including at the telomeres, primarily because of the loss of H3K9me3, resulting in the deregulation of homologous recombination [25]. We have previously shown that Origin Recognition Complex-Associated (ORCA/LRWD1) protein modulates homologous recombination activity by localizing at the ALT-telomeres (Figure 2).

In addition to binding to H3K9me3, ORC and ORCA also associate with H3K27me3, a mark highly enriched on the inactive X-chromosome [112,119] (Figure 2b). The Zhang lab, using an elegant screen to identify factors involved in X-chromosome inactivation, identified several replication factors, including Orc2 and ORCA [119]. They found that Orc2 co-localized or was adjacent to the H3K27me3 focus or the Xist RNA. Further, the loss of Orc2 was found to disrupt silencing on the inactive X, and its impact on inactive X-silencing occurred through the stabilization of HP1a on the inactive X. ORC-ORCA not only binds to the H3K27me3 repressive mark, but also associates with the PRC2 complex and the methyltransferase EZH2 that catalyzes H3K27me3. ORCA functions as a scaffold, and its loss results in the destabilization of methyltransferase protein levels. Equally confusing is the fact that the loss of these methyltransferases disrupts ORCA binding to chromatin, suggesting that these factors are in a complex with one another. It is important to determine whether ORCA is a methyl-binding protein. These observations provide a unified theme that ORC proteins are multi-talented, and their chromatin association is related to their role in replication initiation and gene regulation.

## 7. Conclusions

One overarching question that has persisted and evolved over time is understanding the intricate mechanisms governing the regulation of DNA replication initiation within the context of chromatin. Emerging technologies, such as advanced imaging techniques, single-cell genomics, high-throughput sequencing, and the AI-based analyses of genome-wide datasets [120], need to be leveraged to gain deeper insights into the spatiotemporal regulation of chromatin and the pre-RC.

## Data Availability

This review summarizes published data from numerous labs. No new data were generated or analyzed in this article, so data sharing is not applicable.

## Abbreviations

IDR: intrinsically disordered region; MCM: minichromosome maintenance; ORC: origin recognition complex; PTM: post-translational modification; pre-RC: pre-Replication Complex; ORCA/LRWD1: ORC-Associated/Leucine-rich and WD-repeat-containing- 1.
